# Metal Complexes of a Novel Schiff Base Based on Penicillin: Characterization, Molecular Modeling, and Antibacterial Activity Study

**DOI:** 10.1155/2017/6927675

**Published:** 2017-06-08

**Authors:** Narendra Kumar Chaudhary, Parashuram Mishra

**Affiliations:** Bio-Inorganic and Materials Chemistry Research Laboratory, Tribhuvan University, M. M. A. M. Campus, Biratnagar, Nepal

## Abstract

A novel Schiff base ligand of type HL was prepared by the condensation of amoxicillin trihydrate and nicotinaldehyde. The metal complexes of Co^+2^, Ni^+2^, Cu^+2^, and Zn^+2^ were characterized and investigated by physical and spectral techniques, namely, elemental analysis, melting point, conductivity, ^1^H NMR, IR, UV-Vis spectra, ESR, SEM, and mass spectrometry measurements. They were further analyzed by thermal technique (TGA/DTA) to gain better insight about the thermal stability and kinetic properties of the complexes. Thermal data revealed high thermal stability and nonspontaneous nature of the decomposition steps. The Coats-Redfern method was applied to extract thermodynamic parameters to explain the kinetic behavior. The molar conductance values were relatively low, showing their nonelectrolytic nature. The powder XRD pattern revealed amorphous nature except copper complex (1c) that crystallized in the triclinic crystal system. The EPR study strongly recommends the tetrahedral geometry of 1c. The structure optimization by MM force field calculation through ArgusLab 4.0.1 software program supports the concerned geometry of the complexes. The in vitro antibacterial activity of all the compounds, at their two different concentrations, was screened against four bacterial pathogens, namely,* E. coli, P. vulgaris, K. pneumoniae, and S. aureus,* and showed better activity compared to parent drug and control drug.

## 1. Introduction

Schiff bases containing penicillin and heterocyclic structural units with N, N donor atoms are considered the most prominent research area in the field of coordination chemistry [1–3]. The various donor atoms in them offer special ability for binding metals. The incorporated metals in the lattice of donor atoms of Schiff base change the physiological, morphological, and pharmacological activities of the compounds. The penicillin based Schiff base is of promising research interest owing to the widespread antibacterial resistance of the medical science. Moreover, the revival of research is essential to generate new Schiff base metal complexes with a diverse range of applications. Schiff base complexes have been used as drugs and have valuable antibacterial [4, 5], antifungal [6–8], antiviral [9, 10], anti-inflammatory [11], and antitumor activities [12]. Besides these, they also bear strong catalytic activity in various chemical reactions in chemistry [13] and surfactant activities [14] and as memory storage devices in electronics [15–17]. One of the compounds used to prepare ligand is amoxicillin, a *β*-lactam antibiotic. It is a broad spectrum, semisynthetic penicillin type antibiotic that has potent bactericidal activity against many gram positive and gram negative bacterial pathogens [18]. It takes action against bacteria by preventing them from forming the cell wall and stopping them from growing. In medical science, it has important application for the treatment of bronchitis, ear infection, pneumonia, throat infections, tonsillitis, typhoid, and urinary tract infections. In combination with other antibiotics, it bears potential applications for the successful treatment of many pathogenic infections. However, synthetic modification in amoxicillin by coordination with metal ions of various types has been found to bear enhanced credibility, as documented in several research papers. Cisplatin is the first metal based drug that emerged in the 20th century and enlightened the world as a promising anticancer drug [19]. Since then several research findings culminated the ideas of inclusion of metals in medicine. Many biological molecules containing pyridine moiety as a part of their structural unit bear enzymatic functions as well as the compounds of diverse biological interest. The pyridine derivatives are reported to have herbicidal, fungicidal, and insecticidal activities and also constitute the major core part of biological enzymes, important vitamins, and toxic alkaloids. Its wide applications in agroindustry and as pharmaceutical ingredients in drug discovery are the key points for this research investigation. Nicotinaldehyde (also called pyridine-3-carboxaldehyde) is a class of heterocyclic compound that has pyridine ring and an aldehyde group at meta-position [20]. Among the other pyridine aldehydes, nicotinaldehyde is suitably preferred for the prevention and treatment of* Acne vulgaris*, a kind of skin disease [21].

**Scheme 1:**
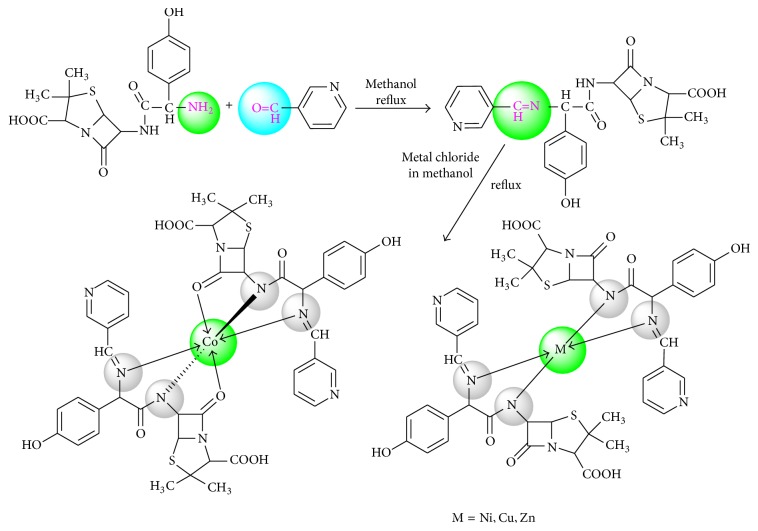
Synthetic scheme for the ligand (HL) and its metal complexes.

In the present paper, we have focused on the synthesis of novel Schiff base ligand, by the condensation of amoxicillin trihydrate and nicotinaldehyde and its four metal complexes with cobalt(II), nickel(II), copper(II), and zinc(II) salts (Scheme 1). The coordination behavior of the ligand towards transition metal ions was fully investigated by various spectral and thermal techniques. The geometry of the complexes was confirmed by energy optimization through MM2 calculation supported in ChemOffice and ArgusLab software program. In continuation of our antibiotic research, we have also evaluated the antibacterial efficacy of ligand and its metal complexes against* S. aureus, E. coli, K. pneumoniae,* and* P. vulgaris* bacteria.

## 2. Experimental Section

### 2.1. Materials

All the chemicals and solvents used were of analytical reagent grade. The title compounds amoxicillin trihydrate and nicotinaldehyde in extra pure form were procured from Duchefa Biochemie, Netherlands, and Spectrochem, Mumbai, India, and used without further purification. Distilled methanol (Qualigen) was used as solvent for the synthesis. The metal salts (Co^+2^, Ni^+2^, Cu^+2^,  and  Zn^+2^ chlorides) (Merck) were used for the synthesis of metal complexes.

### 2.2. Physical Measurements

Elemental microanalysis of the compounds was performed on EURO VECTOR EA 3000 micro analyzer. Melting points of the ligand and its complexes were recorded on an OMEGA melting point apparatus. The pH measurement was done on the Elico-16 pH meter. The infrared (FTIR) spectra of the prepared ligand and metal complexes were recorded on Perkin-Elmer Spectrum version 10.03.06 FT-IR spectrometer that was run as KBr discs in the range 4000–400 cm^−1^. The ^1^H NMR spectra were recorded on Bruker Avance III, 400 MHz spectrometer, using DMSO-d_6_ as solvent. The electronic absorption spectra of the complexes were recorded on single beam microprocessor Labtronics UV-Vis spectrophotometer (LT-290 model) in the range 200–1000 nm in DMSO solvent. EPR-JEOL spectra of the complexes were recorded on JES-FA200 ESR spectrometer with X-band at room temperature. ESI-MS spectra were recorded in positive mode on Agilent Q-TOF mass spectrometer equipped with an electron spray ionization source in the mass range of 200 to 1100. X-ray powder diffraction determinations were accomplished using Bruker AXS D8 Advance X-ray diffractometer with monochromatized Cu-K*α* line at wavelength 1.5406 Å as the radiation source and the measurements were taken over the range of 2*θ* (10 to 70°). The thermal events of the compounds (TGA/DTA) were recorded on a Perkin-Elmer thermal analyzer with a linear heating rate of 20°C min^−1^ in the range of 40–730°C. The surface morphology of the synthesized ligand and metal complexes was analyzed by scanning electron microscopy technique. JEOL JSM-6390 LV scanning electron microscope was used for this investigation.

### 2.3. Synthesis of Ligand (HL)

Amoxicillin trihydrate (2.097 g, 5 mmol) in distilled methanol (30 ml) was stirred under hot conditions for 3 hs. Solubility in methanol was marked at the temperature elevation state. Its pH was adjusted to neutral by adding 0.1 N NaOH solution. Nicotinaldehyde (0.5378 g, 5 mmol) was added slowly to the well stirred amoxicillin trihydrate solution and refluxed under stirring condition for 4 hs. A clear bright yellow solution was left undisturbed for crystallization by slow solvent evaporation process for three days. The resulting solid product was separated, recrystallized with methanol, and dried in desiccator over anhydrous CaCl_2_. The ligand was stored in the airtight vial in the refrigerator till its further use. M. pt. 140°C.* Anal.* C_22_H_22_N_4_O_5_S (454.13): Calcd. C 58.14, H 4.88, N 12.33, O 17.60, S 7.06; Found C 58.21, H 4.81, N 12.25, O 17.57, S 6.95. IR (KBr pellet, selected bands): ${\overset{-}{\upsilon }}_{max}$ = 3303 (b, N-H and O-H str.), 1640 (s, C=N), 1510, 1443 (s, COOH). ^1^H NMR (400 MHz, [D_6_] DMSO):*δ* = 10.122 (s, 1 H, COOH), 9.425 (b, 1H, Ar-OH), 9.094 (s, 1H imine), 8.535–8.864 (m, 4H pyridine ring), 8.241–8.271 (s, 1H NH-amide), 6.718–7.625 (d, C-H aromatic), 1.118–1.562 (C-H methyl) ppm. UV/Vis: *λ*_max_ = 206, 262, 356 nm. ESI-MS, positive: *m*/*z* = 455 [M + H]^+^. Conductivity: Λ_*M*_ = 10.8 *μ*S/cm.

### 2.4. Synthesis of Metal Complexes

#### 2.4.1. Co(II) Complex (1a)

A solution of ligand (HL) (0.454 g, 1 mmol) in 10 ml methanol was stirred for 1 h under warm condition and a solution of CoCl_2_·6H_2_O (0.119 g, 0.5 mmol) in 5 ml methanol was added dropwise with continuous stirring condition. Then after the mixture solution was refluxed for 1 h over water bath with stirring, till blue colored precipitate resulted. The precipitate was filtered from the supernatant liquid, washed with methanol, and dried over anhydrous calcium chloride, yield (65%). M. pt. 285°C.* Anal.* C_44_H_46_CoN_8_O_12_S_2_ (1001.2): Calcd. C 52.74, H 4.63, N 11.18, O 19.16, S 6.40; Found C 52.69, H 4.69, N 11.26, O 19.20, S 6.64. IR (KBr pellet, selected bands): ${\overset{-}{\upsilon }}_{max}$ = 3417 (b, O-H str.), 1633 (s, C=N), 1510, 1443 (s, COOH), 606 (*ρ*_*w*_ H_2_O), 526 (Co-O), 425 (Co-N). UV/Vis: *λ*_max_ = 263, 346–371, 457–488, 549 nm. ESI-MS, positive: *m*/*z* = 1001.2 [M + H]^+^. Conductivity: Λ_*M*_ = 21.8 *μ*S/cm.

#### 2.4.2. Ni(II) Complex (1b)

The nickel complex (1b) was prepared according to the procedure adopted for the preparation of 1a. A solution of NiCl_2_·6H_2_O (0.1188 g, 0.5 mmol) in 5 ml methanol was used for this purpose. The mixed solution of ligand (HL) and Ni^+2^ salt was refluxed for 1 and 1/2 h over water bath which resulted in green colored complex, yield (62%). M. pt. 270°C.* Anal.* C_44_H_42_N_8_NiO_10_S_2_ (964.18): Calcd. C 54.73, H 4.38, N 11.60, O 16.57, S 6.64; Found C 54.55, H 4.59, N 11.59, O 16.45, S 6.44. IR (KBr pellet, selected bands): ${\overset{-}{\upsilon }}_{max}$ = 3337 (b, O-H str.), 1625 (s, C=N), 1513, 1435 (s, COOH), 687 (*ρ*_*w*_ H_2_O), 429 (Ni-N). UV/Vis: *λ*_max_ = 261, 346, 460, 549 nm. ESI-MS, positive: *m*/*z* = 964.18 [M + H]^+^. Conductivity: Λ_*M*_ = 19.9 *μ*S/cm.

#### 2.4.3. Cu(II) Complex (1c)

The copper complex (1c) was also prepared according to the procedure adopted for the preparation of 1a and 1b. A solution of CuCl_2_·2H_2_O (0.085 g, 0.5 mmol) in 5 ml methanol was used for this purpose. The mixed solution of ligand (HL) and Cu^+2^ salt was refluxed for 1 and 1/2 h over water bath which resulted in green colored complex, yield (65%). M. pt. 260°C.* Anal.* C_44_H_42_CuN_8_O_10_S_2_ (969.18): Calcd. C 54.45, H 4.36, N 11.55, O 16.49, S 6.61; Found C 54.52, H 4.49, N 11.63, O 16.55, S 6.73. IR (KBr pellet, selected bands): ${\overset{-}{\upsilon }}_{max}$ = 3379 (b, O-H str.), 1636 (s, C=N), 1512, 1436 (s, COOH), 686 (*ρ*_*w*_ H_2_O), 444 (Ni-N). UV/Vis: *λ*_max_ = 227, 259, 337, 344, 485 nm. ESI-MS, positive: *m*/*z* = 969 [M + H]^+^. Conductivity: Λ_*M*_ = 35.2 *μ*S/cm.

#### 2.4.4. Zn(II) Complex (1d)

The zinc complex (1d) was prepared according to the above procedure and by using Zn^+2^ salt (0.07 g, 0.5 mmol). The mixed solution was refluxed for 2 h over water bath which resulted in light yellow colored complex, yield (57%). M. pt. 250°C.* Anal.* C_44_H_42_N_8_O_10_S_2_Zn (970.18): Calcd. C 54.35, H 4.35, N 11.52, O 16.45, S 6.60; Found C 54.41, H 4.43, N 11.57, O 16.49, S 6.57. IR (KBr pellet, selected bands): ${\overset{-}{\upsilon }}_{max}$ = 3340 (b, O-H str.), 1629 (s, C=N), 1512, 1437 (s, COOH), 657 (*ρ*_*w*_ H_2_O), 415 (Zn-N). ^1^H NMR (400 MHz, [D_6_] DMSO):*δ* = 10.123 (s, 1 H, COOH), 9.425 (b, 1H, Ar-OH), 9.295 (s, 1H imine), 8.534–8.865 (m, 4H pyridine ring), 6.720–7.66 (d, C-H aromatic), 1.118–1.571 (C-H methyl) ppm. UV/Vis: *λ*_max_ = 263, 346 nm. ESI-MS, positive: *m*/*z* = 970 [M + H]^+^. Conductivity: Λ_*M*_ = 5.6 *μ*S/cm.

### 2.5. Antibacterial Susceptibility Test

The antimicrobial potency of the synthesized compounds was done by assaying antibacterial activity study. The experimental portion of the study was accomplished in the laboratory of the Department of Microbiology at Mahendra Morang Adarsh Multiple Campus, Biratnagar. The compounds (HL and 1a–1d) were tested in vitro by standard Kirby-Bauer paper disc diffusion method against some gram positive and gram negative human pathogenic bacteria [12, 22, 23]. The recommended NCCLS guideline was followed for the study [24]. Well sterilized filter paper discs of 5 mm size (Whatman-model) were used as antibiotic assay discs for testing of compounds. The discs were loaded with test compounds at two different concentrations (100 and 50 mcg/mcl in DMSO) under UV laminar flow to reduce bacterial contamination [25]. The loaded discs were dried in the laminar flow chamber by blowing hot air through hair drier. Sterilized nutrient agar media were carefully poured in the Petri plate and kept in rest for few hours in the sterilized zone for solidification. Fresh bacterial culture, revived before injection, was swabbed on the media and the loaded discs were stuck over it. One disc soaked with DMSO was used as the solvent control and amikacin (30 mcg/disc) was used as positive control. Inoculated plates were incubated at 37°C for 24 hs, and the diameter of the zone of inhibition was measured by antibiogram zone measuring scale [26].

## 3. Results and Discussion

### 3.1. Physical Characterization

The physical properties and the microanalytical data of the ligand (HL) and metal complexes (1a–1d) are summarized in the experimental section. The analytical results show (1 : 2) metal ligand ratio, that is, ML_2_ type. The color change from ligand to metal complexes is in support of metal ligand interaction which is further reinforced by conductivity and pH change. The ligand (HL) was soluble in methanol. The complexes were soluble in DMSO and DMF. The nickel complex (1b) was found to be hygroscopic. The suggested molecular formulae of the ligand (HL) and metal complexes (1a–1d) have been achieved by microanalytical results in combination with various spectral techniques. The experimental molar conductivity data of HL and metal complexes was found in the range of 5.6–35.2 *μ*S/cm and suggests their nonelectrolytic nature. The pH of ligand and complexes was almost in the neutral range.

### 3.2. Spectral Characterization

**Figure 1:**
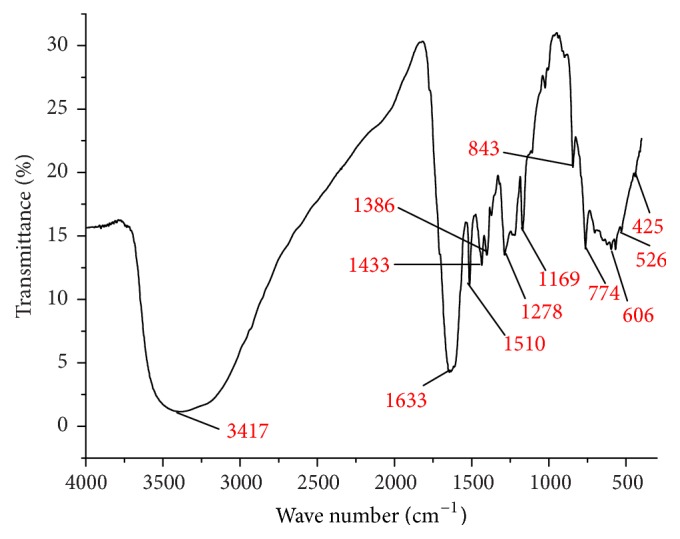
IR spectrum of cobalt complex (1a).

The formation of HL was confirmed by ESI mass spectrometry, which showed peaks at *m*/*z* = 455, attributable to [M + H]^+^. The FTIR spectrum is also in line with the proposed structure of HL, with characteristic stretching vibrations at 1640 cm^−1^ assignable to azomethine group [27]. A broadband with absorption maximum of 3303 cm^−1^ is possibly due to collapse of N-H and O-H stretching peaks. Other significant strong bands at 1510 and 1433 cm^−1^ for HL are attributed to *ν*(COOH) asymmetric and symmetric stretch. The ^1^H NMR spectrum of HL executes a sharp singlet at 9.09 ppm corresponding to azomethine proton. On complexation, *ν*(C=N) stretching band for HL has shifted to lower absorption frequency of 1633 cm^−1^ (1a), 1625 cm^−1^ (1b), 1636 cm^−1^ (1c), and 1629 cm^−1^ (1d), indicating the coordination of azomethine nitrogen atom to the metal ion [28]. This lower frequency shift of azomethine group in the complexes is due to the decrease in electron density and force constant of the metal with the azomethine nitrogen lone pair. In all the complexes, FTIR absorption bands corresponding to *ν*(O-H) execute in the range 3337–3417 cm^−1^ relative to 3303 cm^−1^ for HL. The complexes exhibit *ν*(COOH) stretching vibrations at the equivalent positions of the ligand, suggesting their noncoordination with the metal centers. The formation of cobalt complex (1a) was confirmed by ESI-MS peak at *m*/*z* = 1002, attributable to [M + H]^+^. The well resolved IR band at 3417 cm^−1^, for complex (1a), corresponds to the *ν*(O-H) stretching vibration (Figure 1) [29]. The evidence of bonding in 1a is also shown by the observation of new bands in the lower frequency regions at 425 and 526 cm^−1^ characteristic to *ν*(Co-N) and *ν*(Co-O) stretching vibrations that are not observed in the IR spectrum of ligand. The less intense IR band at 606 cm^−1^ is assignable to bending vibration of two lattice water molecules of the outer sphere region. The observed molecular mass of nickel complex (1b) was evidenced by ESI mass spectrum peak value at *m*/*z* = 964, assignable to molecular ion peak. The formation of this complex was verified by FTIR spectroscopy, where specific bands are observed at 1625 cm^−1^  *ν*(CH=N), 3337 cm^−1^  *ν*(O-H), 429 cm^−1^  *ν*(Ni-N), and 687 cm^−1^ for outer sphere lattice water molecules. The copper complex (1c) executes a strong azomethine band at 1636 cm^−1^ which has undergone a negative shift by 4 cm^−1^ relative to that of the free ligand. The other significant FTIR bands are observed at 3379 cm^−1^  *ν*(O-H), 444 cm^−1^  *ν*(Cu-N), and 686 cm^−1^ for outer sphere lattice water molecules. The formation of the complex 1c is further evidenced by the ESI-MS peak at *m*/*z* = 970, attributed to [M + H]^+^. The positive ion ESI mass spectrum showed peaks at *m*/*z* = 971 for zinc complex (1d), attributed to [M + H]^+^. Its formation was strongly evidenced by FTIR and ^1^H NMR spectral data. The strong azomethine band at 1629 cm^−1^  *ν*(CH=N) for this complex has shifted by 11 cm^−1^ towards a lower wave number relative to that of the free ligand, indicating metal coordination with azomethine nitrogen. The metal nitrogen coordination is further evidenced by a sharp peak at 415 cm^−1^ in the FTIR spectrum of 1d. The ^1^H NMR spectrum is also consistent with the suggested structure. The downfield shift of ^1^H NMR signal for azomethine proton from *δ* 9.094 ppm for ligand to *δ* 9.295 ppm for zinc complex (1d) also supports coordination of the azomethine nitrogen to the zinc(II) ion. Two doublets observed at *δ* 6.718–7.625 for HL and *δ* 6.72–7.66 ppm for zinc complex 1d are attributed to aromatic ring protons. The methyl protons of amoxicillin moiety in both HL and 1d appear as a singlet peak in the region of *δ* 1.118–1.562 ppm. Amide NH proton for HL executes signal at 8.241–8.271 ppm, which is absent in the spectrum of 1d, and this confirms the coordination of amide N-atom with metal center via deprotonation [30]. In the spectrum of HL, the signal due to carboxylic proton appears at *δ* 10.122 ppm, which is still present in the ^1^H NMR spectrum of zinc complex (1d).

### 3.3. Electronic Absorption Spectra and Magnetic Moment Measurement

The electronic absorption spectrum of ligand (HL) displays high energy bands in the ultraviolet region at 206 and 262 nm, corresponds to *π* → *π*^*∗*^ transitions of the aromatic and pyridinium ring, and, at 356 nm, corresponds to *n* → *π*^*∗*^ intraligand charge transfer band with the involvement of C=N group [31]. However, the additional bands in the higher wavelength region are observed in the complexes which signify metal ligand coordination. The cobalt complex (1a) exhibits two distinct bands in high wavelength region of the spectrum at 457–488 nm and 549 nm. The former band is assignable to ^4^T_1g_(F) → ^4^T_1g_(P) and latter band indicates ^4^T_1g_ → ^4^A_2g_ transition, confirming its octahedral geometry [32]. The magnetic moment value (4.75 BM) further supports this geometry. The high energy bands for this complex are observed at 263 and 346–371 nm, assignable to *π* → *π*^*∗*^ and *n* → *π*^*∗*^ LMCT transitions, respectively. The electronic absorption spectrum of nickel complex (1b) displays d-d transition band at 460 nm assignable to ^1^A_1g_ → ^1^B_1g_ transition along with the bands in the low wavelength region at 261 and 346 nm [33, 34]. The diamagnetic nature of this complex is suggestive of the complete distortion of octahedral geometry and confirms its square planar geometry. The magnetic moment value (1.82 BM) and electronic absorption spectrum of paramagnetic copper complex (1c) exhibit absorption band in the high wavelength region at 485 nm, attributed to ^2^T_2g_ → ^2^E_g_ transition which is suggestive of tetrahedral geometry [35]. Other high energy bands for this complex are observed at 227 and 259 nm for *π* → *π*^*∗*^ transition and 337 and 344 nm for *n* → *π*^*∗*^ LMCT transition. The zinc complex (1d) displays an absorption band at 346 nm assignable to the LMCT transition, compatible with tetrahedral geometry, and this is further supported by its diamagnetic nature and absence of d-d band, due to its complete d^10^ electronic configuration.

### 3.4. TGA/DTA Studies

The TGA/DTA curves for the complexes were carried out within the temperature range from room temperature to 700°C with the linear heating rate of 20°C/min in the nitrogen atmosphere. Correlation of the thermal events at elevated temperatures with kinetic parameters provides useful physicochemical information of the compounds. Thermo gravimetric analysis is one such important instrumental technique to observe thermal changes with respect to increase in temperatures [36]. The computed thermal decomposition data in Table 1 are in good agreement with the suggested microanalytical data. The following findings have been achieved in our research analysis.

**Figure 2:**
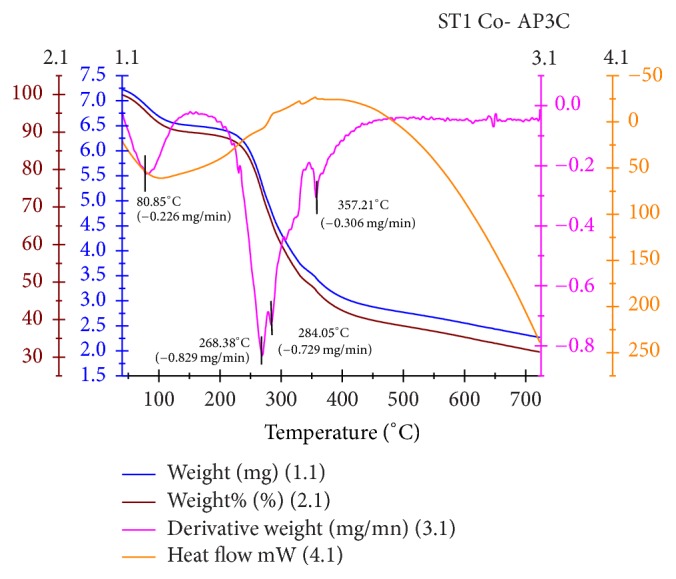
Thermogram of cobalt complex (1a).

The thermogram of cobalt complex (1a) (Figure 2) exhibited four decomposition steps in the temperature range of 50–380°C. The first decomposition step in the temperature range of 50–110°C with % mass loss of 4.826% (0.226 mg) is assignable to the loss of two lattice water molecules from the outer sphere [37, 38]. The second and third decomposition steps with % mass loss of 25.923% (0.829 mg) and 33.837% (0.729 mg) in the temperature range of 241–273°C and 279–300°C have considered the loss of organic ligand moiety. The last decomposition step with % mass loss of 52.247% (0.306 mg) represents a complete loss of ligand from the complex in the temperature range of 338–380°C, leaving cobalt oxide as stable residue. The nickel complex (1b) exhibited thermal decomposition in two distinct steps. The first step with % mass loss of 5.6% (0.153 mg) is assignable to the loss of outer sphere lattice water molecules in the temperature range of 44–113°C. The exothermic peak with 30.86% (0.484 mg) mass loss in the temperature range of 232–403°C is attributed to loss of ligand moiety. The thermograms of other two complexes 1c and 1d are complement to the analyzed data of 1a and 1b. The first step decomposition in 1c and 1d occurred in the temperature range around 45–107°C with T_DTG_ 76.79 and 75.46°C and this again suggests the loss of two lattice water molecules. The final thermal decomposition step in all the metal complexes is noticed above 400°C, which is indicated by the formation of the horizontal TG curve. This step interprets the formation of stable metal oxide residue. 

*Kinetic Parameters*. The thermal dehydration and decomposition of the complexes were studied by using an integral method applying a very popular Coats-Redfern method [39, 40]. The thermodynamic activation parameters of decomposition processes are essential to describe the thermal stability as well as the nature and rates of thermal decomposition of the complexes. These parameters are evaluated graphically by plotting of data based on Coats-Redfern relation in the following form:

(1) $$\begin{array}{c} \ln\left[\;-\frac{\ln\left(1-\alpha \right)}{T^{2}}\;\right]=\ln\left[\;\frac{AR}{\beta E^{*}}\;\right]-\frac{E^{*}}{RT}, \end{array}$$

where *α* represents the decomposition fraction at temperature *T* K and *β* denotes linear heating rate (*dT*/*dt*). *E*^*∗*^ and *A* denote the activation energy and Arrhenius preexponential factor, respectively. *R* represents gas constant. Molding the equation for the straight line (*y* = *mx* + *c*), a linear plot of left side versus 1/*T* of Coats-Redfern equation gives a straight line, whose slope *E*^*∗*^/*R* furnishes activation energy parameter and the preexponential factor (*A*) can be determined from the intercept. The other thermodynamic parameters such as entropy of activation (Δ*S*^*∗*^), enthalpy of activation (Δ*H*^*∗*^), and free energy of activation (Δ*G*^*∗*^) have been calculated by using the following relation:

(2) ΔS∗=Rln⁡AhkBTΔH∗=E∗−RTΔG∗=ΔH∗−TΔS∗.

The computed data of thermodynamic activation parameters of various decomposition steps of the metal complexes are listed in Table 2. In the present work, the plot of left hand side of Coats-Redfern equation versus 1000/T in all the decomposition steps of all complexes shows a best fit for first-order reaction kinetics [41]. The high and increasing values of activation energy in the subsequent steps of all the complexes reflect high thermal stability, which may be due to covalent bond character. The entropy of activation value of first decomposition step in all the complexes is negative, which indicates nonspontaneous dehydration reaction process. Most of this value of other steps is positive and infers the dissociation character of decomposition [42]. This also attributes more ordered activated state than the reactants. The positive Δ*G*^*∗*^ values of all the complexes justify the nonspontaneous nature of decomposition steps. The enthalpy of activation values (Δ*H*^*∗*^) in most of the decomposition steps is positive which reveal endothermic processes. However, this nature also depends upon the value of other thermodynamic activation parameters. The computed data of correlation coefficient (*r*) obtained from the graphical plot reflect a good fit of the data with linear function [43, 44].

### 3.5. XRPD Study

**Figure 3:**
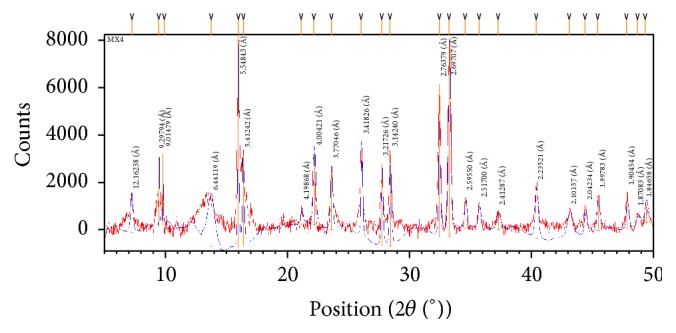
X-ray diffractogram of copper complex (1c).

Single crystal growth of the synthesized compounds was unsuccessful, so their crystallinity was established by X-ray powder diffraction study. The ligand (HL) and complexes (1a, 1b, and 1d) were found amorphous. The crystal structure of copper complex (1c) was worked out by its well resolved crystalline peaks (Figure 3), which crystallized in a triclinic crystal system with P1 space group. The diffractogram of this complex registered 22 reflection peaks in the range of (2*θ*) 0 to 50° with maxima at 15.974° with corresponding *d* spacing value of 5.584 Å. The cell dimensions* a*  (6.2282 Å),* b*  (109390 Å),* c*  (20.3388 Å),*α*  (63.1585°),*β*  (113.5723°),* and γ*  (64.269°) are in good agreement with the refined triclinic crystal system. The unit cell volume of this compound was calculated to be 747.4131 Å^3^ with FOM 31. The details of crystallographic data are summarized in Table 3. The particle size was calculated from Scherer's formula *α* = 0.9*λ*/*β* cos⁡*θ*, where*λ* is the wavelength, *β* is the full-width half maximum of the characteristic peak, and *θ* is the diffraction angle of the* hkl* plane [45, 46]. The average particle size 69.34 nm suggests its nanocrystalline nature.

### 3.6. EPR Analysis

**Figure 4:**
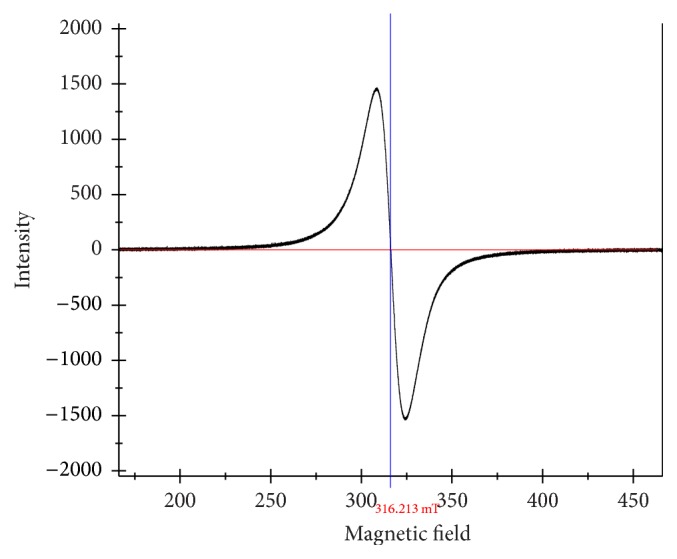
EPR spectrum of copper complex (1c) at room temperature.

The solid state X-band EPR spectrum of the copper complex (1c) was recorded at room temperature under the frequency 9447.606 MHz with no marker lines used and center line at 316.213 mT. The standard lines that are used in EPR model are of Mn, which has been omitted in the graph. The EPR spectrum of complex provides useful information about the metal ion environment within the complex. The highly symmetrical EPR spectrum of copper complex (1c) (Figure 4) delivered a single isotropic signal with *g*_||_ value of 2.18 and *g*_⊥_ value of 2.08 [47]. The absence of poorly resolved hyperfine signal may be attributed to the considerable exchange coupling interaction of the Cu^+2^ ions in the complex. The order of splitting factors *g*_||_ > *g*_⊥_ > 2.0023 clearly indicates the localized unpaired electron in the *d* orbital of Cu^+2^ ion and is characteristic of axial symmetry [6, 48]. The calculated *g*_av_ value is 2.11 whose deviation from the free electron (2.003) is due to covalent character of the metal ligand bond. This fact is further supported by *g*_||_ value less than 2.3. The value of exchange coupling interaction parameter “*G*” = 2.25 is less than 4 and suggests considerable exchange interaction in the complex [49]. All these parameters are in support of tetrahedral geometry of copper complex (1c).

### 3.7. SEM Analysis

**Figure 5:**
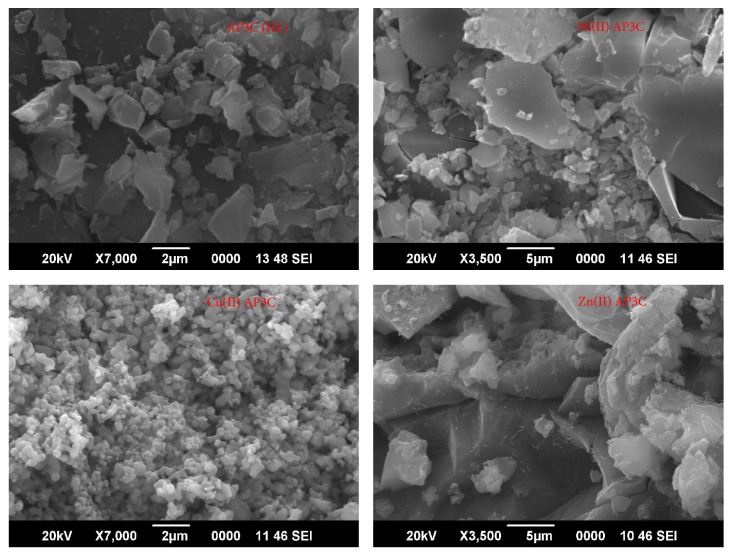
SEM micrographs of HL, 1b, 1c, and 1d.

The metal coordination to ligand significantly changes the surface morphology of the complexes and this was investigated by SEM analysis. The SEM micrograph of ligand (HL) and metal complexes are shown in Figure 5 and the differences are seen in surface morphology of the metal complexes due to changes in the metal ions. The SEM micrograph of ligand demonstrates nonuniform platelet-like structure with variable lateral dimensions [50]. Moreover, inhomogeneous matrix with broken ice-like structure has been observed in the SEM micrograph of nickel complex (1b). The SEM micrograph of copper complex (1c) displays agglomerated morphology with small sized grains scattered in homogenous matrix and gives the appearance of coral-like structure. In the SEM micrograph of zinc complex (1d), small sized particles crumbled together to give rock-like structure with somewhat cotton-like appearance.

### 3.8. Molecular Modeling

**Figure 6:**
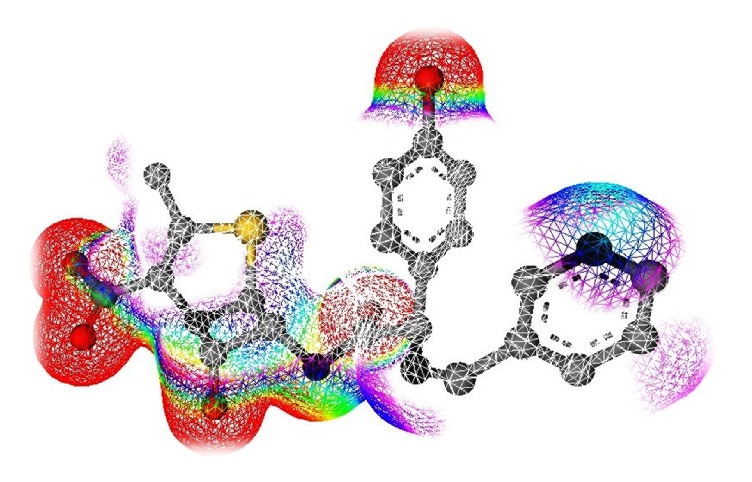
Electrostatic potential mapped electron density surface of HL.

**Figure 7:**
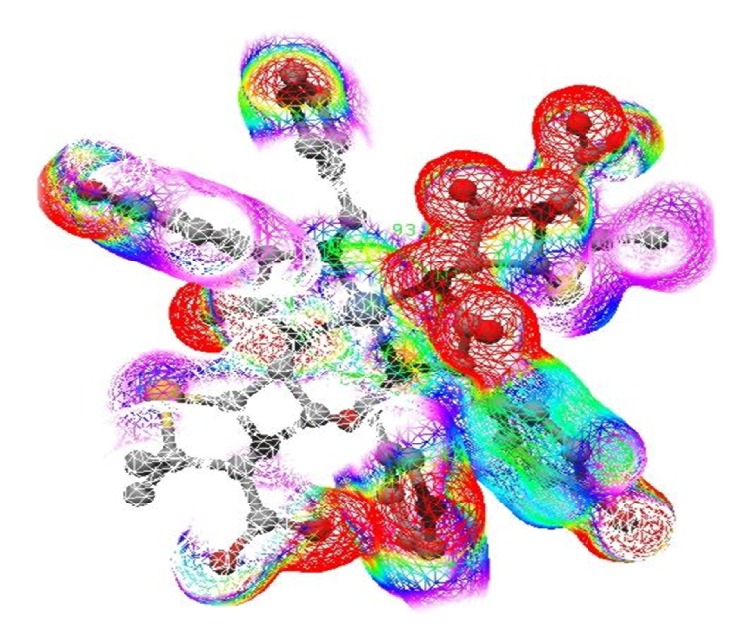
Electrostatic potential mapped electron density surface of nickel complex (1b).

The computational study of the compounds furnishes a clear idea about the three-dimensional arrangement of different atoms in the molecules. The energy optimization of the ligand (HL) and metal complexes (1a–1d) was done by Universal Force Field (UFF) technique with minimum RMS gradient 0.100, supported in ArgusLab 4.0.1 version software [51, 52]. The details of the bonding and energy parameters optimized by molecular modeling calculations of the metal complexes are depicted in Table 4. For ligand, single point energy calculation with Hamiltonian AM1 revealed final SCF energy and heat of formation, −132288.8349 and 45.0637 kcal/mol, respectively [53]. After the geometry optimization by molecular mechanics (UFF) technique, the final geometrical energy of HL has been reported to 139.2725 kcal/mols. On ESP mapped electron density surface of HL (Figure 6), red color indicates the highest electron density region which is around O-atom. The second highest electron density region is around an azomethine N-atom which is shown by mixed green and yellow colors. This is the region for stability of coordinated metal ions and supports its linkage with azomethine N-atom. It seems clear that the coordination with O-atoms is restricted due to greater electronic repulsion and field obstruction. In the nickel complex (1b), the high electron density (Figure 7) around the coordinated azomethine N-atom, indicated by red color, is in favor of its proposed geometry. Similar study and computational data of the complexes (1a to 1d) are in good support of their proposed structures.

### 3.9. Antibacterial Activity Study

**Figure 8:**
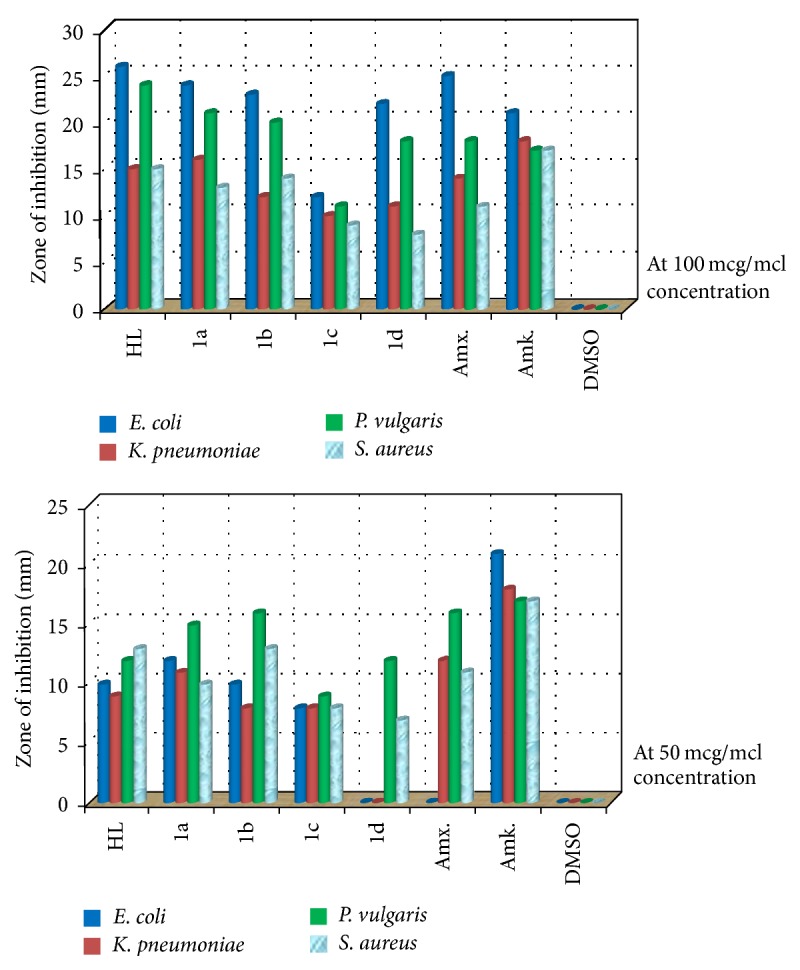
Bar graph of antibacterial evaluation study.

The antibacterial efficacy of the ligand (HL) and metal complexes (1a–1d) was tested against* S. aureus, E. coli, K. pneumoniae,* and* P. vulgaris* bacteria. The antibacterial results are presented in the bar graph (Figure 8). Two different concentrations (100 and 50 mcg/mcl) of the compounds have been selected for antibacterial assay. The results suggest enhanced antibacterial activity of the ligand (HL) and metal complexes (1a–1d). The compound (1c) showed little activity against all the bacterial pathogens, compared to ligand and other metal complexes. The ligand bears activity, even greater than parent drug amoxicillin and control drug amikacin at higher concentration. This higher activity of ligand is possibly due to interference in the normal cell process of organism caused by the formation of hydrogen bond through the azomethine group with the active center of cell constituents [54]. Further, the uncoordinated heteroatom of pyridine moiety also contributes to microbial growth inhibition. Moreover, the complexes deliver better antibacterial activity at their higher concentration. Precise observation reveals that the compounds are less active against* S. aureus* and more active against* E. coli* and* P. vulgaris* bacteria. This enhanced activity of the complexes may be attributed to chelation of Schiff base with metal ions that provide stability and more susceptibility against the bacterial pathogens [55, 56]. It has been suggested that the structural components possessing additional (C=N) bond with nitrogen and oxygen donor systems inhibit enzyme activity due to their deactivation by metal coordination. This permits their efficient permeation through the lipid layer of organisms and destroys their activity [6].

## 4. Conclusion

The novel ligand (HL) and the metal complexes (1a–1d) were successfully synthesized. The ligand can complex the metal ion via N donor atoms. The electronic absorption spectral analysis in combination with ESR data revealed octahedral geometry for cobalt complex (1a), square planar geometry of nickel complex (1b), and tetrahedral geometry for both copper complex (1c) and zinc complex (1d). Several spectral data nicely support the above concerned geometry of the complexes. Furthermore, the metal complexes were screened in vitro for antibacterial assay. Based on the results of this study of synthesized compounds, it has been concluded that the ligand bears greater potency than amoxicillin and control drug amikacin. The complexes 1a, 1b, and 1d were even highly active against all the bacterial pathogens at their higher concentration; however the copper complex (1c) was less active than others. This greater activity might be due to azomethine linkage and heteroatoms present in these compounds.

## Figures and Tables

**Table 1:** Thermal decomposition data of metal complexes.

Comp.	Step	TG range (°C)					DTA	
		Δ_*m*%_ *found*	*T*_*i*_	*T*_*f*_	*T*_DTG_	Mass loss	*T*_dta_	Peak
**1a**	1	4.826	50	110.2	80.85	−0.226	103.68	Endo
	2	25.923	241.63	276.73	268.38	−0.829	—	—
	3	33.837	279	300	284.05	−0.729	—	—
	4	52.247	338	380	357.21	−0.306	334.9	Endo

**1b**	1	5.601	44	113	85.91	−0.153	105.33	Endo
	2	30.861	232	403	278.16	−0.484	382.91	Exo

**1c**	1	2.699	49	105	76.79	−0.100	111.23	Endo
	2	8.814	148	163	159.86	−0.395	—	—
	3	21.635	187	246	237.13	−0.310	—	—
	4	42.395	293	390	326.01	−0.244	385.92	Exo

**1d**	1	3.047	46	107	75.46	−0.121	115.75	Endo
	2	18.233	230	287	260.99	−0.491	339.24	Endo
	3	36.441	310	410	330.41	−0.238	365.88	Exo

**Table 2:** Kinetic and thermodynamic parameters of metal complexes.

Comp.	Step	*r*	*A *(s^−1^)	*T*_max_ (K)	*E* ^*∗*^ (kJ/mol)	Δ*S*^*∗*^ (J/K·mol)	Δ*H*^*∗*^ (kJ/mol)	Δ*G*^*∗*^ (kJ/mol)
1a	1	−0.98495	5.62 × 10^6^	353.85	58.36	−117.92	55.4719	97.19789
	2	−0.99209	5.06 × 10^19^	541.38	229.22	126.559	224.718	156.202
	3	−0.99052	1.19 × 10^36^	557.05	403.95	18.3647	399.319	389.089
	4	−0.99343	1.14 × 10^22^	630.21	288.1474	170.339	282.907	175.525

1b	1	−0.99453	7.054 × 10^6^	358.91	59.62	−40.236	56.634	71.075
	2	−0.9887	1.35 × 10^13^	551.16	160.7139	35.4267	160.288	140.762

1c	1	−0.99401	4.064 × 10^7^	349.79	63.4725	−110.3698	60.564	64.191
	2	−0.99698	10.06 × 10^51^	432.86	447.68	765.803	444.080	112.594
	3	−0.99643	5.98 × 10^11^	510.13	129.453	−24.716	125.2115	137.819
	4	−0.99604	6.55 × 10^5^	599.01	93.1345	−140.1638	92.6438	176.603

1d	1	−0.99448	5.89 × 10^6^	348.46	57.8981	−117.3976	55.00	95.908
	2	−0.99491	2.92 × 10^15^	533.99	176.7582	45.523	172.318	148.00
	3	−0.99118	7.64 × 10^5^	603.41	96.5845	−103.320	91.567	153.911

**Table 3:** X-ray powder diffraction data of copper complex (1c).

Peak number	2*θ*	*θ*	Sin⁡*θ*	Sin^2^⁡*θ*	*h* ^2^ + *k*^2^ + *l*^2^	*hkl*	*d*	FWHM	% int.	*a* in nm
1	7.2685	3.63425	0.06338	0.004017	1	0 0 1	12.16238	0.2244	16.00	61.22
2	13.7483	6.87415	0.11968	0.014323	1	0 1 0	6.44119	0.6731	21.90	20.37
3	15.9738	7.9869	0.13894	0.019304	2	1 1 0	5.54843	0.0935	100.00	146.64
4	16.3780	8.189	0.14243	0.020286	10	1 0 −3	5.41242	0.1496	42.23	91.65
5	21.1607	10.58035	0.18361	0.033712	9	1 2 2	4.19868	0.2244	6.47	61.102
6	22.2011	11.10055	0.19253	0.03706	11	1 −1 −3	4.00421	0.2244	33. 02	61.102
7	23.5966	11.7983	0.20446	0.041803	1	1 0 0	3.77046	0.1870	28.72	73.323
8	26.0686	13.0343	0.22553	0.050863	6	1 1 2	3.41826	0.1309	44.73	104.747
9	27.7288	13.8644	0.23962	0.057417	4	0 2 0	3.21726	0.1496	38.35	91.654
10	28.4032	14.2016	0.24533	0.060186	10	1 3 0	3.14240	0.1496	42.24	91.654
11	32.3941	16.19705	0.27894	0.077807	20	2 0 −4	2.76379	0.1122	70.54	122.205
12	33.2184	16.6092	0.28584	0.081704	40	2 0 −6	2.69707	0.2057	91.73	66.657
13	34.5584	17.2792	0.29702	0.088220	13	2 0 −3	2.59550	0.2244	13.44	61.103
14	35.6718	17.8359	0.30629	0.093813	2	1 −1 0	2.51700	0.2244	8.91	61.103
15	37.2666	18.6333	0.31951	0.102086	20	2 4 0	2.41287	0.3739	7.05	36.671
16	40.3520	20.176	0.34490	0.11895	80	0 4 8	2.23521	0.2617	23.89	52.394
17	43.0415	21.52075	0.36683	0.13456	9	1 −2 −2	2.10157	0.4487	11.08	30.5585
18	44.3550	22.1775	0.37747	0.14248	30	2 5 1	2.04234	0.2244	11.90	61.103
19	45.3976	22.6988	0.38588	0.14890	69	2 1 −8	1.99783	0.2244	15.90	61.103
20	47.7559	23.87795	0.40478	0.16384	25	3 4 0	1.90454	0.1870	16.97	73.324
21	48.6714	24.3357	0.41211	0.16983	58	3 0 −7	1.87083	0.2991	5.79	45.843
22	49.3094	24.6547	0.41714	0.174	18	3 3 0	1.84658	0.2736	7.79	50.116
							Average particle size			69.3474

**Table 4:** Selected bond lengths and bond angles of metal complexes.

Complex	Atoms	Bond length (Å)	Bond energy (Kcal/mol)	Atoms	Bond angle	Bond angle energy	Final geom. energy
1a	N(3)-Co(34)	1.957	273.796	N(3)-Co(34)-N(20)	90.00	300.46	349.2538 (Kcal/mol) (0.556 au)
	N(20)-Co(34)	1.972	267.453	N(3)-Co(34)-N(35)	90.00	304.025	
	Co(34)-N(35)	1.957	273.796	N(3)-Co(34)-N(52)	90.00	300.46	
	Co(34)-N(52)	1.972	267.453	N(3)-Co(34)-O(104)	90.00	273.401	
	Co(34)-O(104)	1.964	244.913	N(3)-Co(34)-O(113)	90.00	273.401	
	Co(34)-O(113)	1.964	244.913	N(35)-Co(34)-N(20)	90.00	300.46	
				N(52)-Co(34)-N(20)	90.00	296.982	
				O(104)-Co(34)-N(20)	90.00	270.214	
				O(113)-Co(34)-N(20)	90.00	270.214	
				N(35)-Co(34)-N(52)	90.00	300.46	
				N(35)-Co(34)-N(104)	90.00	273.401	
				N(35)-Co(34)-N(113)	90.00	273.401	
				N(52)-Co(34)-O(104)	90.00	270.214	
				N(52)-Co(34)-N(113)	90.00	270.214	

1b	N(2)-Ni(33)	1.870	313.824	N(2)-Ni(33)-N(19)	90.00	344.228	324.5763 (Kcal/mol) (0.517 au)
	N(19)-Ni(33)	1.885	306.275	N(2)-Ni(33)-N(34)	90.00	348.473	
	N(34)- Ni(33)	1.870	313.824	N(2)-Ni(33)-N(51)	90.00	344.228	
	N(51)- Ni(33)	1.885	306.275	N(34)-Ni(33)-N(19)	90.00	344.228	
				N(51)-Ni(33)-N(19)	90.00	340.090	
				N(34)-Ni(33)-N(51)	90.00	344.228	

1c	N(2)-Cu(33)	2.016	181.007	N(2)-Cu(33)-N(19)	109.47	158.764	373.488 (Kcal/mol) (0.595 au)
	N(19)-Cu(33)	2.031	176.938	N(2)-Cu(33)-N(34)	109.47	160.587	
	N(34)-Cu(33)	2.016	181.007	N(2)-Cu(33)-N(51)	109.47	158.764	
	N(51)-Cu(33)	2.031	176.938	N(34)-Cu(33)-N(19)	109.47	158.764	
				N(51)-Cu(33)-N(19)	109.47	156.977	
				N(34)-Cu(33)-N(51)	109.47	158.764	

1d	N(2)-Zn(33)	1.888	164.142	N(2)-Zn(33)-N(19)	109.47	193.167	352.3697 (Kcal/mol) (0.561 au)
	N(19)- Zn(33)	1.903	160.260	N(2)-Zn(33)-N(34)	109.47	195.503	
	N(34)- Zn(33)	1.888	164.142	N(2)-Zn(33)-N(51)	109.47	193.167	
	N(51)- Zn(33)	1.903	160.260	N(34)-Zn(33)-N(19)	109.47	193.167	
				N(51)-Zn(33)-N(19)	109.47	190.879	
				N(34)-Zn(33)-N(51)	109.47	193.167	
